# Humanitarianism and the Quantification of Human Needs: Minimal Humanity

**DOI:** 10.1017/S1816383124000389

**Published:** 2024-10-10

**Authors:** Joël Glasman

**Keywords:** humanitarian principles, impartiality, humanity, humanitarian action, humanitarian organizations, human needs, international humanitarian law, Beyond the Literature

## Abstract

*In this iteration of the* Revi*ew*’*s* “*Beyond the Literature*” *series, we have invited Joël Glasman to introduce his recent book* Humanitarianism and the Quantification of Human Ne*eds, before then posing a series of questions to Bertrand Taithe, Léa Macias, Dennis Dijkzeul, Andrea Behrends and William Anderson. Bertrand Taithe is Professor of Cultural History at the University of Manchester in the United Kingdom. Léa Macias is an anthropologist focusing on digital humanitarianism in the Middle East, currently working as an Evaluation Officer for the French Development Agency. Dennis Dijkzeul is Professor of Organization and Conflict Studies at Ruhr University Bochum, Germany. Andrea Behrends is Professor of Social and Cultural Anthropology at Leipzig University, Germany. William Anderson is the Executive Director for Sphere based in Geneva*.

*The* Review *team is grateful to all five discussants, and to Joël, for taking part in this engaging conversation*.

## Joël, why did you write this book? What are its key messages? What would you hope to add if the book, published in 2020, were being written today?

**Joël Glasman:** This book came out in response to the data frenzy of the early 2010s. At the time, there was a lot of excitement about humanitarian statistics. Some claimed that new technologies like big data, remote sensing and machine learning would make humanitarian aid truly universal. There was talk of a “revolution” – the United Nations [UN] Secretary-General himself talked about a “data revolution” – and of the advent of “evidence-based humanitarianism”. Humanitarian workers were being asked more and more to produce numbers, aggregate data and use statistics. Reports like the UN Office for the Coordination of Humanitarian Affairs [OCHA] *Global Humanitarian Overview* were basically just a set of statistics. But the discourse on statistics wasn’t just about technology. It wasn’t just about making humanitarian aid more efficient. It was also a moral discourse. It was about humanitarian principles, about making aid more just, more equitable and more impartial – as if statistics were the only sound way to talk about our common humanity.

When I started my research, there were two different approaches to explain the rise of humanitarian statistics. The first was an optimistic one, which drew heavily on Max Weber’s idea that bureaucracy is based on knowledge production. It argued that humanitarian statistics were the result of the bureaucratization of aid, the professionalization of careers, the rationalization of expertise, and so on. There was some truth to it, because in fact, a process of bureaucratization has happened in the humanitarian sector over the last twenty years. However, this approach didn’t really address the side effects of quantification, or the limits of it, or the mistakes that can be made. The second approach, a more pessimistic one, drew largely on Michel Foucault’s work on neoliberal governmentality. In this theory, statistics are not neutral. They’re a tool used by big donors to control the humanitarian sector indirectly, through things like evaluation, benchmarking, notation, logframes and bookkeeping. There was an excellent literature on this, from scholars like Didier Fassin, Jennifer Hyndman, Michel Agier and Béatrice Hibou.

But both interpretations partly ignored the internal workings of humanitarian organizations. “Minimal humanity” suggested a third interpretation, informed by science and technology studies and pragmatic sociology. It looked at how the people involved in humanitarian work had decided to value certain techniques over others. It also looked at how these organizations had combined technology and morality in a particular way. This approach doesn’t deny that larger processes like bureaucratization and neoliberalism are important, but it argues that humanitarian organizations do have a certain degree of autonomy and agency in defining their priorities.

Obviously, a lot has changed since the book was published. For starters, there’s been a lot of new research on humanitarian aid and statistics. For instance, there’s been new research on datafication of refugee camps, on humanitarian statistics and the media, and on the connection between data production and racial biases. Scholars like Kristin Sandvik, Brendan Lawson, Mamane Souley Issoufou, Crystal Biruk, Nehal Bhuta and the contributors to this discussion have made significant contributions to the literature. Second, the geopolitical context has changed. In our post-coronavirus world, the optimism of the 2010s is definitely gone. The wars in Ukraine, Sudan, Ethiopia and Gaza have shown us more urgent problems like lack of access, war crimes and the targeting of humanitarian workers. We don’t know where the humanitarian system is going, but we do know that the way we collect data is going to change. The claim that statistics calculated by high-performance machines will ensure impartiality in the delivery of humanitarian assistance doesn’t seem very convincing when countries fail to condemn war crimes perpetrated by their allies.

## Bertrand, Léa, Dennis, Andrea and William, do you share the book’s central thesis that the quantification of human needs has shaped the humanitarian sector and changed the practices, aims, targets and scope of humanitarian aid?

**Bertrand Taithe:** Yes, I tend to agree with some of the key arguments on this book that a desire to quantify – perhaps more stridently and urgently formulated than the reality of quantification – has long been at the heart of a range of sometimes contradictory urges in humanitarian aid and, incidentally, development programmes. Where perhaps we tend to disagree is in the degree to which this desire to quantify has genuinely impacted delivery and whether humanitarians ever had the means of their ambition. In our study of UN data, we concluded that there was hubris in this desire and that we could add a desire to enumerate to other forms of narrative rhetoric to explain and justify humanitarian work.^1^ As part of a vast array of tools to shape the storytelling of humanitarian aid, represent needs and justify expenditures, figures – reflecting actual detailed accounting or sublimated large figures – have always played a central part in the representation of work done, work to be done, needs met and needs that will never be met. This is not to undermine the notion of quantification or the central point of this book, but merely to historicize it further.

**Léa Macias:** Absolutely. As Glasman manages to prove very eloquently in his book, the quantification of human needs has shaped the humanitarian sector towards quantification as the sole proof of the impartiality of organizations and the justification of intervention. The quantification of needs has become an entire span of humanitarian organizations’ activities, both at the HQ and field levels. Entire departments have organized themselves around the datafication process of needs. Yet even more than the scope, quantification has changed the relations between humanitarian workers and the so-called “beneficiaries”. Data collection is now a new way of communicating between humanitarian organizations and the people they intend to assist. It has changed the relationship to the “field” and therefore the day-to-day work of humanitarian workers. Decision-making processes based on needs also mean that the person taking the decision is often remote from the field, solely looking at spreadsheets. These developments have also changed the perspective on humanitarian spaces; the remoteness of some places can be made even further by the illusion of data being collected and published on an online platform.

**Dennis Dijkzeul:** Yes, I do. Without quantification of human needs, the current humanitarian system would cease to function. Humanitarians would have a hard time communicating – and agreeing – with each other; the Humanitarian Needs Overviews and Humanitarian Response Plans (nowadays often combined into Humanitarian Needs and Response Plans [HNRPs]) that help set priorities for humanitarian action would not be possible; the cluster coordination system would barely function; and donors would often not know how to assess and fund projects. The same holds true for the Integrated Food Security Phase Classification System and many other humanitarian data tools and techniques.

The great contribution of Joël Glasman’s book is that it explains in a nuanced manner the history of how this process of quantification has taken place. Glasman does a wonderful job explaining how concepts (in particular, changing conceptions of need), classification (legal, economic, etc.), artefacts (e.g., mid-upper arm circumference [MUAC] tape) and standards (Sphere) contribute to quantification. Experts who use these concepts and tools often know and discuss their limitations, but at the moment their outcomes become aggregated, the quantitative data on needs look increasingly unassailable. Put differently, decisions taken in humanitarian headquarters or donor capitals, or media reports, are based on such data, but the limitations become neglected. These data are then also used to compare the needs and effects of aid across different crises and societies, without sufficiently taking into account the extent to which such data is comparable.

Importantly, Glasman critiques critical theory. In this respect, he shows that humanitarian organizations do not just follow neoliberal management approaches, and nor are they just “deploying a kind of biopolitical domination that targets territories, bodies, and populations, and thereby shapes people’s needs”.^2^ Instead, he indicates that the tools for quantification display a certain autonomy on the part of humanitarian organizations. At the same time, the quantitative data does not show how the humanitarian system itself functions.

The irony of this process is that you can easily criticize quantification, but you cannot easily do without it. The question becomes: how we can enhance the quality of quantification? To answer this question, we must go beyond quantification to a critique of the humanitarian system. There are several aspects to this critique.

First, quantification always starts with a qualitative question. Put differently, thinking about what one wants to know always starts with an open, non-quantitative question. In this sense, quantitative and qualitative approaches are not opposites, but rather are complementary. How these approaches are being used and whether they complement each other depends on decisions by humanitarian donors, organizations and clusters.

Second, quantification of needs is always a reduction. In particular, contextual knowledge is left out. This brings up the question of what is more important for humanitarian action: knowledge of the technical aspects of aid provision (which is often quantified), or knowing the local context (cultural preferences and traditions, different types of actors and their coping mechanisms, power relations and priorities, local perceptions of the international humanitarian organizations and their activities,^3^ perhaps forms of clientelism or corruption, and so on)? Of course, we need both, but their balance is hard to establish. Currently, the humanitarian system tilts towards technical skills on the assumption that they can be transferred from one crisis to another. Often context can be grasped better through qualitative research and requires a long-term field presence of humanitarian actors. To give just a small example on disability inclusion, humanitarian organizations increasingly collect gender-, age- and disability-disaggregated data, which is good, but it needs to be complemented by more qualitative disability barrier and enabler analysis. The timing of such an analysis is also important. If it comes too late, in the autumn, for example, it will not impact the HNRPs much. Later, political power play during implementation also influences disability inclusion. Contextual knowledge should receive more attention in the humanitarian system, which brings me to the next point.

Third, if we look at the system, we should also discuss its broader set-up and incentives. Unsurprisingly, donor funding plays a crucial role. It does not just determine which types of actors and activities are financed, but also the (short) length of funding cycles and rapid staff turnover, as well as the types of risk management, due diligence, reporting and capacity-building that are implemented. Donor governments will not do capacity-building of local organizations but will delegate it to UN organizations and non-governmental organizations [NGOs]. The clusters function in their own way. If a local organization becomes active in a cluster, it helps when its representative speaks English or another UN language and knows some of the international organizations active in the cluster. Let’s take a certain Ukrainian front-line organization as an example. Before the full-scale invasion in 2022, it worked as a group of volunteers with older people in the region. Once the war broke out, it informally started to help these people with humanitarian support and house visits. Making such changes and working in or close to a war zone is already a major challenge; simultaneously adapting to the international humanitarian system, and all its funding, risk management, due diligence, capacity and reporting requirements, is well-nigh impossible, despite all the talk about localization. It is actually surprising that some Ukrainian organizations have nevertheless succeeded in doing so. Hence, quantification is part of a broader process that can either exclude such local organizations or force them to adapt to the international aid system. In response, we can ask: how can the international system adapt to local actors? At the very least, long-term relationships and qualitative research can facilitate reaching such small organizations, of which there are many in Ukraine and elsewhere. This could also be facilitated by long-term and/or more direct funding.

**Andrea Behrends:** Joël Glasman’s book clearly and very elaborately shows how definitions of “human needs” have come to be based on quantitatively generated data that are “indispensable for decision making”^4^ in relation to humanitarian intervention. Your question is if this quantification has changed practices within the humanitarian sector. I would say that rather than being shaped by the quantification of human needs, I understand Glasman’s book to demonstrate that it is the humanitarian sector that has shaped the ways in which quantifications have become the basis of classifying people as “people in need”, and that the indicators that enable quantification and generalization of the human condition in relation to “needs” also originate in the humanitarian sector. These classifications determine who receives what form of assistance. This is to say that changes brought about by quantification are an inherent part of the transformation within the humanitarian process itself. So what is most interesting for me is the processual and differential part of quantifications.

Glasman points out that the entitlement to protection and assistance only comes after classification, for instance, through the agents working with the Office of the UN High Commissioner for Refugees [UNHCR]. He also shows that although these quantifications seem to apply to every human globally, the indicators are applied differently in countries that are considered rich, poor or in-between. One example for the differentiation in standards of quantification that he introduces is the *Sphere Handbook*’s Minimum Standards in Humanitarian Response. To define a situation as a “humanitarian crisis”, these standards use the number of deaths per 10,000 people, but this number is not the same everywhere. As Glasman writes about the *Handbook*’s third edition in 2011:

Thus, starting in 2011, a humanitarian crisis would no longer be defined universally as a situation provoking more than one death per 10,000 per day, but rather 1.07 in sub-Saharan Africa, versus 0.46 in South Asia, 0.15 in Latin America, and 0.03 deaths per 10,000 people per day in “industrialized countries.” In other words, to be considered a “humanitarian crisis” in Africa, a catastrophe now must result in 35 times as many victims as in Europe.

Concerning what is at stake for “minimum standards”, Glasman quotes Satterthwaite to critically remark: “if universal standards are in fact not universal, the very concept of ‘minimum standards’ begins to lose meaning”.^5^

What I consider particularly compelling about this work is that it can show how both the method of quantifying data and the classifications that result from quantified data have historically changed or have constantly been adapted to new situations. In other words, the way the need for help has been defined has always had something to do with how humanitarian aid organizations have positioned themselves at different times.

**William Anderson:** Firstly, I would like to appreciate the diligent work of Joël Glasman in researching this book. Since publication, the downstairs apartment mentioned on page 126 of the book^6^ has been vacated, and the numerous archive boxes have been winnowed and whittled down to all but the most important papers. Joël’s published work therefore represents an important record for Sphere, and I am glad to share that Joël and I have been in communication since I started at Sphere, and he is welcome back anytime.

Prior to the *Sphere Handbook* there was little common language or consistency of terms in the humanitarian sector. This is one of the key aspects of how Sphere has shaped how humanitarians frame any given response, for, as I will explain in more detail later on, Sphere is not about quantifying needs as such, it is about respecting everyone’s right to life no matter the situation. When designing and planning humanitarian projects it is necessary to have a consensus on common metrics so that agencies are not working in the dark; Sphere’s guidance therefore provides a yardstick which if not already written would be in great demand now.

Contrary to some detractors’ assumptions as described in Joël’s book, the founders of the Sphere Project did not intend to standardize the sector. In fact, their primary aim was a focus on dignity and to incorporate a *To Kill a Mockingbird* “Atticus Finch” moment each time principled humanitarian actors interacted with crisis-affected people. To this end, a quick review of all agencies’ communications, including values, straplines and reports, will somewhere likely include the concept of dignity, and the quantification of needs as a vehicle to supporting this has been successful in shaping the sector. Humanitarian action should be asking a foundational question: what level of quality of assistance would we expect, and, given the power dynamics in play, how can humanitarians be accountable to the very people they are assisting? The Humanitarian Charter, Core Humanitarian Standard and Minimum Standards channel both assistance planning and implementation action into this overall framework approach.

As such, and given the perpetual hand-wringing in the sector over how much more we should be doing to further accountability to affected people, my conclusion is that the “quantification of human needs” has not significantly changed our engagement practice at community level – but it has shaped the sector in terms of the primary focus on dignity as entrenched in Article 1 of the Universal Declaration of Human Rights: “All human beings are born free and equal in dignity and rights.” This aspect is fundamental to then appreciating project-level indicators and their role in driving quality, which in turn goes some way to respecting every individual, and their rights, in who they are as a person. Agencies, clusters and donors alike have so much more data driving quality programming, and often use the *Sphere Handbook* and other Humanitarian Standard Partnership handbooks either to set indicators or to achieve them. Positive impact is dependent largely on context. The *Sphere Handbook*, therefore, with the qualitative Minimum Standards and accompanying contextualized Key Actions with quantitative Key Indicators, should not be viewed as a threat or alarm bell which “judges” humanitarian actors who are unable to meet certain standards due to the context, such as Gaza or Sudan right now. Sphere’s resources are instead helpful and supportive guidance to enable agencies to implement and advocate for quality operations that respect the dignity of crisis-affected people. If the sector were to give up on quality and just do what it can without trying to achieve a minimum standard at some point, then the sector is giving up on dignity and our shared humanity – the very cornerstone of humanitarianism. It is also important to note that each individual is different and that good-quality humanitarian assistance will be one of many factors when a person considers their feelings of dignity.

Sphere is relevant in preparedness and recovery as well as response contexts, for although the Minimum Standards are focused on life-saving assistance, they are applicable in short-, medium- and long-term responses. It’s worth repeating that the *Handbook* should be contextualized wherever it is used – the Key Actions and Key Indicators are not universal. The Minimum Standards provide a strong rights-based framework for local actors to lead humanitarian response and ensure proper contextualization of work being done. Global humanitarian qualitative standards are not a binding set of rules but are benchmarks to influence and inform good humanitarian practice. As the introduction to the *Sphere Handbook* notes, “[t]he degree to which agencies can meet standards will depend on a range of factors, some of which are outside their control”. There are standards that will not apply in all contexts, and the way in which those which are relevant are met will necessarily differ from situation to situation. In this regard, the contextualized use of standards furthers the localization agenda.

It is also worth highlighting that regarding localization and nexus, Sphere is grounded in Articles 6 and 8 of the Code of Conduct of the International Red Cross and Red Crescent Movement [Red Cross Code of Conduct]: “We shall attempt to build disaster response on local capacities”, and “Relief aid must strive to reduce future vulnerabilities to disaster as well as meeting basic needs”.

## Humanity is central to principled humanitarian action. Does access to data that quantifies humanitarian needs – including communicating their sometimes massive scale – help humanitarians further this principle? Does it help the public connect with the humanity of those experiencing humanitarian crisis?

**Dennis Dijkzeul:** Yes, but only imperfectly. First, as Glasman highlights, quantification leads to a change in the conceptualization of humanity. Needs have become the lowest common denominator of humanity – a minimal humanity that can be measured but leaves out how people, including those in need, are or should be connected with each other. As Glasman notes:

The question at stake here is the very nature of “humanity”: A long-distance society tied through mutual obligations, or a loose bond between people sharing mere “human nature”? In the 1940s, the idea of a vital minimum was closely associated with a claim for social justice and equality, even on a world scale. Now, humanitarianism has become a “prisoner of the contemporary age of inequality”, to borrow Samuel Moyn’s expression.^7^

Glasman explains well how certain aspects of need are left out in quantification. As indicated in my answer to the first question, we do not get a full picture of individuals, organizations or societies. As a result, the humanitarian system itself is left out of the equation. It is not critically scrutinized.

Nevertheless, the massive scale of current needs can often only be communicated and made commensurable in numbers. But it should also be clear that numbers are not the most emotive factors – pictures and personal stories make it easier to connect with people affected by crises. Don’t forget that Henry Dunant’s *A Memory of Solferino*^8^ is more a tearjerker than a quantitative evaluation report.

All in all, humanitarian action remains an imperfect offering.^9^ In a world with limited – and for many crises, declining – resources, it is unlikely that humanitarian action can go beyond minimal humanity. In this respect, quantification is a double-edged sword. It highlights certain aspects but hides others.

**Léa Macias:** Not so much humanity, because data turns away from the personal stories, the realities of the sufferings and the lives of the people about whom data is collected. Just as Glasman compares Dunant’s words to the ones produced in humanitarian reports, data quantifying needs help defining the target of the humanitarian assistance. In that sense it can also serve the same purpose of connecting the public with the necessity of the humanitarian intervention. Quantification of needs can of course help leverage large-scale gap funding exercises, such as the Humanitarian Needs Overview conducted by OCHA, as the data produced about the needs is meant to be shared with governments, donors etc. as its main audience. Narratives about the people suffering from a specific crisis are also being used as a powerful tool by humanitarian organizations. Yet the data itself regarding “human needs” is not so much about enhancing humanity than justifying the impartiality of a worldwide intervention by humanitarian organizations.

**William Anderson:** Sphere is about rights-based, principled, quality and accountable humanitarian action, and calls for the dignity of all crisis-affected people to be respected. It was started in 1997 by impassioned aid workers who wanted to improve the quality and accountability of emergency response. With this goal in mind, they framed the Humanitarian Charter and identified a set of Minimum Standards to be applied contextually in all humanitarian crises.

Sphere’s flagship publication, the *Sphere Handbook*, is one of the most widely known and internationally recognized sets of humanitarian principles and minimum standards and puts the rights of disaster-affected populations to life with dignity, protection and assistance at the heart of humanitarian response. It is not a rule book and is more than a handbook – it represents a vibrant community of principled and concerned people.

The Minimum Standards represent decades of practice by thousands of leading experts. The “consensus” of the Standards is based on collective evidence of good practice and a commitment to further improvement as we move forward. Because of contextual differences in crises, it is difficult to show that Sphere has universally improved humanitarian response. However, we do have evidence of the effectiveness of the Standards in improving life-saving efforts in numerous specific contexts. Beyond that, it is important to note that consensus itself – particularly across such a large and diverse set of actors – underscores the relevance, importance and effectiveness of the Sphere Minimum Standards.

The Humanitarian Charter formulates the right to humanitarian assistance. It was not intended as a signatory commitment, but to complement the Red Cross Code of Conduct and the UN Charter. The founders did not intend to standardize assistance as such, but to define the minimum content of contextualized aid entitlement (sufficient quantity and quality of goods and services) in order that people’s dignity could always be respected. This is an important point regarding quantification: Sphere is not built upon numbers, as the Minimum Standards are qualitative; rather, it is built upon the Humanitarian Charter and human rights. This is why restricting the guidance to mere numbers misses the whole point of the *Handbook*.

**Andrea Behrends:** Considering especially the massive scale of global human needs makes relying on quantification a necessity for humanitarian action. Unlike in the early days of humanitarian intervention, it is no longer possible to base interventions on personal experience and individual cases. There is a need to rely on quantifications. Quantification becomes problematic when it is faced with the need to decide who will get access to aid and who will not. This is a process that is called “triage”, from the French term *trier* – sorting, selecting, inspecting. Triage provides the answers to questions such as: who should be treated first? Who should generally be prioritized? Who should be helped right now? Who must wait? These questions concern our feeling of being human and being treated as such. Triage is used in hospital emergency wards, in reference to COVID patients, or in the context of refugee relief. It is used anywhere where the allocation of scarce resources is at stake, particularly when neither the local communities to which people flee during wars or other catastrophes nor the regional or national governments of States are able to shoulder this provision on their own.

But while humanitarian action can refer back to quantifiable data during triage, the public perceives triage as questionable and inhuman. Moreover, what Glasman’s book’s historical perspective shows is how relying on quantified data is deeply tied to global political and economic interests. This often remains invisible to the public. The UNHCR’s crisis interventions that are at the centre of the book’s case studies therefore lie not so much in providing protection – as one might think – but rather, as Glasman points out, in the agency’s near-monopoly over the “modes of classification”. Whoever defines the classifications influences the fates of people. I find this to be an extremely important insight, especially when it comes to the question of decolonizing humanitarian aid. Understanding historical shifts in how classifications are applied might also help publics to connect to the humanity of people experiencing crisis as well as to the aid approach.

**Bertrand Taithe:** The reliability of humanitarian data was always a moot point.^10^ Denouncing famines always entailed some detailed calculation of need, for instance, combined with the impossibility of accounting precisely for all needs. Refugee work also tends to be based on debatable estimates of population, as Oliver Bakewell has argued.^11^ Using proxies and defining needs according to indicators, clinical data or market prices always raised the possibility of inflated figures which might undermine credibility.^12^ Of course, this danger to credibility is only institutional if it does not impact resources – denouncing a famine which is “merely” a major subsistence crisis does not directly impact the recipients of food if the food gets to be distributed. When in 2005 Médecins Sans Frontières denounced a famine which other observers had not seen^13^ – when humanitarians sought to define the terms of famine in Somalia^14^ – the calculations were far from simple and “needology” proved to be a more impressionistic approach, elaborating from incomplete data and seeking best estimates in lieu of figures. When it becomes a tragedy is when the calculation of needs does not lead to an adequate response. The obvious danger of calculating figures is of course the need to compete with other bad news that is recurring with disarming regularity. This is the power of very large numbers: the casualties in the tens of thousands, the victims in their millions, arguably promote an inflation arising from habituation to sufferings, as denounced by Susan Sontag.^15^ The endless reiteration of figures can lead to a dehumanization, and the shock of re-cognition may indeed operate against the principle of humanity itself. The whole notion of thresholds for famine and crisis points has long operated on the basis of a calculation of what might be normality is fixed in time and does not match the development of societies, as Fabrice Weissman argues.^16^ The epidemiological data used to define an acute crisis may create false notions of the returned-to normality as well. Ultimately it operates on profoundly shocking notions of the unequal value of human life. In many ways these calculations may remain buried below the headline figures and in the processes of establishing minimal norms, as Glasman’s book shows.

## Like humanity, impartiality is also key to principled humanitarian action. How can we understand impartiality in the context of quantified needs? Are numbers the most efficient guardian of humanitarian impartiality?

**William Anderson:** Humanity and impartiality are the goals of humanitarian action. Humanity aims to reduce suffering, protect life and promote respect for human beings. Impartiality prioritizes the focus of the response on those with the greatest unmet need and without adverse distinction. This equitable nature demands that contextual specificities and data, including numbers, are required in order to determine which community or group is indeed the most vulnerable and most in need.

The principles of neutrality and independence are organizational, means to an end, *modus operandi*. As such they are not individual morals or goals or values in and of themselves, but ways of working to enable the collective. Applying these principles can help facilitate the achievement of the goals of humanity and impartiality by ensuring the acceptance of all parties to the conflict that assistance is necessary and relevant, and thereby improve the safety and security of crisis-affected people and humanitarians alike.

Clarity on what the humanitarian principles are and how they may be applied is important. If we do not know what the principles are, we can, for example, avoid thinking about them through fear of ignorance or impostor syndrome, or we may discuss them at cross-purposes or unintentionally compromise them, all of which may impact access and humanitarian outcomes. Uncertainty about the humanitarian principles also creates hesitancy to discuss how they apply to a humanitarian response with teams, partners and counterparts due to the risk of public embarrassment. A common understanding of the four humanitarian principles as two ethical stances and two operational instruments is required for the sector to retain and promote the principles. This is why an overly simplistic approach, grouping them as one set, is unhelpful.

**Bertrand Taithe:** The notion of impartiality that is referred to in the process of quantification is rooted in ideas of distributive fairness. This is not the only way of apportioning foodstuffs or resources; rationing in wartimes and besieged cities followed other criteria of usefulness in military or compassionate allocations. The notion of triage, which operates sometimes counter-instinctively at the expense of those suffering the most to favour the ones likely to benefit the most from medical treatment, is another instance of quantification which may not seem fair, at first sight, even when it is applied consistently. The notion that all should get the same treatment in times of emergency is never applied in this sense. Those processes which rest on evaluations and some degree of quantification – but perhaps very complex and unspoken quantification – are not necessarily absolutely impartial, or rather, as Glasman argues, impartiality itself needs to be questioned in relation to quantification processes. Quantification makes palatable or bearable a humanitarian response which can never meet every need to the extent that one might wish. The hubris of humanitarianism is to imagine that it could ever meet all the needs it may measure. Glasman’s book makes some of these processes more evident and shows how the history of these processes impacts on their formulation. It does not claim that quantification is, in itself, a guardian; rather, it is merely a cognitive tool among others which may seem, at some distance, less arbitrary than others. Quantitative evaluation mechanisms contain, however, deeply seated in their calculus, a number of choices which remain otherwise unspoken.

**Andrea Behrends:** My first answer would be that just like the notion of need, the notion of impartiality is not easily generalizable. But to relate it to Glasman’s book, I would like to turn to his example of the measuring of body parts. One of his prominent examples is the MUAC band that measures a child’s mid-upper arm circumference to discover malnutrition. It was used by humanitarian aid agencies during the Central African Republic’s war and the ensuing refugee situation in Cameroon, where Glasman did research. By tracing the history and the band’s current use (at the time of writing the book), he found that the band is supposed to impartially discover children who do not have enough to eat, but there are malnourished children who show other traits of malnutrition than the mid-upper arm circumference, and they would not be covered by this measure.

In this case, humanity is reduced to the need to get special food to survive. For the case of Central Africa, Glasman shows that children’s arms were measured and their level of malnutrition was determined, along with the health status of the entire family. If a displaced family was deemed “healthy” by this measurement, they received cornmeal and rice as food rations. When they had “moderately acutely malnourished” children, they were given a special supplemental food called Plumpy’Sup. Only those children who were classified as “severely acutely malnourished” were sent to the hospital for treatment.

From a historical perspective, Glasman’s work shows that the bases upon which such decisions are taken are by no means objective or neutral – that is, they do not represent the “view from above” as science often likes to boast. On the contrary, these measurements originated in certain contexts, specifically the Nigerian Civil War in Biafra in 1969. Glasman’s book shows that the threshold, namely the measurement of the arm circumference of a 2-year-old child, became smaller and smaller between 1969 and 2009. This is due to the fact that different measurement methods were set as the standard. Today, the arm circumference has been reduced from 13 cm to 11.5 cm. Concerning such decisions that are hidden from public view, the book’s criticism is important: Glasman underlines that in an ever more affluent world, the determination for acute malnutrition has even been lowered.

**Léa Macias:** As Glasman demonstrates, numbers have become the guardian of humanitarian impartiality. Indeed, humanitarian organizations are being put under a lot of pressure to demonstrate their need for action in a particular context. Therefore, data quantifying the needs is a powerful tool to make the case for a humanitarian intervention. Yet numbers are still a political and social construct from the way the standards are set, translated into questionnaires, to the teams recruited to collect the data, etc. What you can collect, where you can collect it, and how, shapes the quantification process itself. It means political contexts still shape the level of response and therefore quantification of needs itself cannot be seen as the sole justification for the impartiality of humanitarian organizations.

**Dennis Dijkzeul:** Impartiality implies providing aid based on needs, regardless of nationality, race, gender, religious belief, class or political opinions. Glasman describes how the idea of non-discrimination has over time been complemented – or perhaps even taken over – by measurement, or better, by the quantitative tools to measure needs.

Let’s not forget that quantitative data can help show, however imperfectly, how high needs are and the extent to which they are being addressed. Much critical theory on humanitarian actions tries to uncover hidden assumptions and power relations in humanitarianism, but looking at such domination often lacks a sense of proportion, because it can leave out the health benefits, the number of people fed, or the survival rates that humanitarian action also brings about. Better numbers can help provide such a sense of proportionality. Ironically, improving quantification can also help address its critics.^17^

In addition, Glasman’s book inspires us to look more broadly at humanitarian practices and ask what they show and what they hide. We can also criticize the humanitarian principles in this respect. One way of summarizing Glasman’s argument is that numbers always have political consequences. Some are visible, some become hidden. The same holds true for the humanitarian principles. The principles are explicit normative claims: on a secular right to intervene (humanity) based on human needs alone (impartiality). Humanity and impartiality are thus justifications for humanitarians to become active in a crisis, be it an armed conflict or a natural disaster. Put differently, they help set the goals of humanitarian action. Together with independence (no impact by donors on the crisis) and neutrality (provision of aid has no impact on the crisis), impartiality is also an operative concept, maintaining (or pretending) that actual humanitarian action will only address needs and will have no impact on the crisis. Just as with quantification, however, the principles do have actual (political) impacts that their normative claims tend to hide. This becomes more visible when we shift our focus from normative claims to cause-and-effect chains in a multi-actor environment, where many actors – warlords, (corrupt) government officials, private enterprises, donor agencies, traditional and religious leaders, and so on – pursue their own interests and (would like to) use or abuse humanitarian action for their own ends. As a result, humanitarian impact can differ considerably from humanitarianism’s normative goals and claims.

Table 1 shows how explicit normative claims tend to hide implicit causal claims.^18^ To summarize this table, just as with quantification, the quality of the actual application of the principles is crucial. Just as with quantification, the principles also hide some of the politics of aid, which paradoxically allows humanitarian action to continue functioning in complex and challenging environments based on the necessary fiction that they do not influence the crisis. Good-quality humanitarian action is possible, but it requires great effort to make quantification or the principles work.

## One critique of quantification of needs in the humanitarian sector is that it reiterates Eurocentric and colonial structures – including because it privileges those contexts about which reliable data is available. How would you respond to this critique?

**Bertrand Taithe:** The illusion here is to over-emphasize the reliability of one culture of data over another. The denunciation of Eurocentrism, in a sense, mirrors those who glorified European quantifications of the world. The colonial empires and colonialism were not consistent producers of reliable data or users of that data. They produced arguably some knowledge, but also a great deal of ignorance and indifference^19^ – the data arising from famines in the colonial setting was often poor and mirrored the great indifference of the rulers towards their colonial subjects.^20^ Data production always reflects active power, but also depths of neglect. The lack of reliable information that can effect actual change in the West is absolutely blatant for some underprivileged communities in Europe. Actual effectual quantification tends to be less common than is claimed, and very few troubled contexts can enable reliable quantitative data collection. The events in Haiti in 2010 showed the inability to account exactly for a major disaster or to calculate precisely how much response to events had taken place. War zones, migration sites of transient presence, and areas that are under-researched due to security risks can all create spaces of lesser quantification.

**Andrea Behrends:** My knowledge about the use of data and where they come from to determine interventions in the humanitarian sector is too limited to give an informed answer to this question. But, based on my own ethnographic work on displacement and aid in the Chad–Sudan borderlands, particularly during and since the war that began in 2003, there is no denying that decisions about where and on what scale to intervene are also driven by global political events – or, as you mention, Eurocentric values and structures.

For instance, the currently ongoing brutal war in Sudan does not gain much attention, and significantly less humanitarian intervention than twenty years ago, and this might be due to international interest in declaring this war a crisis that calls for intervention – or not. I can only speculate about the reasons, but if we compare the massive scale of the intervention in 2003, with around 100 aid organizations and international NGOs, to almost no intervention now, we might also take a look at the circumstances of deciding to intervene in both time periods. In 2004, Colin Powell, then US secretary of State, declared the war in Darfur to be the “first genocide of the 21st century”. On the one hand, with the 1994 genocide in Rwanda still in vivid memory, this declaration was said to enable interventions in order to prevent more gruesome killing. At the same time, Powell’s statement also turned attention away from some flaws in relation to the United States’ “war on terror” that followed the 9/11 terrorist attacks, when it appeared to be the case that the United States’ assumptions about extensive stockpiles of chemical and even nuclear weapons could not be proved.

Whatever its intention, Powell’s declaration of genocide resulted in massive intervention, both military and humanitarian. Aid agencies and international military troops entered the region, changing not only the social but also the political and economic situation in the borderlands for quite a number of years to come. In contrast to that, the currently ongoing war in Sudan, which is also fought in Darfur and is raging worse than before, remains almost completely invisible on the global level.

**William Anderson:** If the *Sphere Handbook* had been viewed as Eurocentric, it would have long since been consigned to the dustbin. Neither the *Sphere Handbook*, nor the Humanitarian Standards Partnership, is about the quantification of needs *per se*. When the Minimum Standards are contextualized by whoever is using the guidance into Key Actions and Key Indicators, quantification is brought to bear, enabling locally owned, principled humanitarian action. Sphere has around seventy Focal Points around the world, not one of whom has ever raised a concern that the guidance is colonial or Eurocentric. On the contrary, they are Sphere Champions precisely because they take ownership of the guidance in their country or region, and the independent organization Sphere India is a great example of this.

**Dennis Dijkzeul:** The critique is only correct to a very limited extent, and it does not offer an alternative. Quantification indeed taps into a deep vein of Western thought,^21^ but there is no reason why it is solely a Western, Eurocentric or colonial concept. Mathematics did not originate in Europe. Stating that quantification privileges certain contexts is useful only when it leads to calls for higher quality and better use of data, which can help to contextualize and improve humanitarian action. In fact, the critique in this question reminds me of the old colonialist argument that “science and education are not for the natives”.

Empirically, it is also increasingly untrue. Look at the increase in data collection in most crises in this world, or the enthusiasm for “big data”. Crucially though, Glasman is rightly sceptical of such enthusiasm. Again, we should discuss the quality and uses – and therewith, the consequences – of this data, which leads to a critique not just of qualitative and quantitative data in the humanitarian system, but also of the functioning of the system itself.

**Léa Macias:** This critique is also because of the way data is being collected, analyzed and shared. It is collected in some of the most remote places in the world, but then appears online, on various platforms, with very little accountability to the people who spent time and opened their homes to answer the questions. Quantification through the datafication of “people in need” is part of an extractive process, as recent research has demonstrated.^22^ A way to alleviate this bias in the quantification of needs would be to adopt a localized approach to data collection and analysis. This would empower local actors to produce information regarding the situation they are facing. Building trust around the data produced at the local level could also empower local organizations in the delivery process of humanitarian assistance.

## What has changed since the book was published in 2020? What, in your view, should future work on the topic of what the author calls “needology” address?

**Andrea Behrends:** Obviously, a lot has changed since the book’s publication. With the COVID-19 pandemic, the practice of triage entered global consciousness. People suddenly experienced or at least could imagine how it must be, within their own close family, to be in a situation of triage where there had been more or less sufficient hospital facilities before. As many hospitals were reaching their maximum capacity, and the respiratory technology that saved lives could only be given to some and not others, who would be the ones to be saved? The questions that plague humanitarian aid agencies in their operations within catastrophic situations in the global South suddenly lay on our doorsteps in Berlin, Rome and New York. Looking at academic or institutionally based work about human need, I would agree with Glasman that this work needs to find a way to take struggles for equality into consideration. Applying humanitarian standards based on quantification might silence such struggles. In Glasman’s words,

[i] ndividuals are seen to have needs that are not linked to social inequalities or power relations, thus implying that the responsibility for a person’s suffering ultimately relies on individual responsibility. Humanity is thought of as an aggregate entity. Humanitarian expertise considers “persons in need” to be individuals who are autonomous, independent, and interchangeable.^23^

Again quoting Margaret Satterthwaite, Glasman notes that the issue of power remains “depoliticized”.^24^

**William Anderson:** As the humanitarian sector has focused significant effort on accountability to affected populations in the past few years, aid quality has largely taken a back seat. It is essential that the quality of humanitarian assistance comes back into the forefront alongside accountability, and that all efforts to respond to crises are grounded in humanitarian principles and minimum standards. Without quality of assistance there can be no assurance of dignity. Respect for the humanity of each person affected by crisis – and “affected” often means traumatized, grieving and devastated – is diminished every time quality is shelved.

Standards provide a common framework for planning and implementation of coordinated and quality emergency assistance. Standards in WASH [water, sanitation and hygiene], food security and nutrition, shelter and health are not being met in some of the world’s most visible and forgotten crisis zones. The more time that passes until these standards are met, the more suffering and death there will be. Unsafe and insufficient water, starvation, overcrowded homes and destroyed hospitals are a certain recipe for humanitarian catastrophe and reflect a failure of humanity.

Finally, a reminder that context is key: if the Minimum Standards cannot be met due to contextual challenges, there should be in place an advocacy and operational plan to meet and exceed them when the context improves. The *Sphere Handbook* is as useful in these contexts as it is in less extreme and severe situations.

**Dennis Dijkzeul:** It would be interesting to know whether new conceptualizations of need will arise. In all likelihood, these will be associated with changing ways of collecting and analyzing data on needs.

Over the last few years, the use of both quantitative and qualitative data in the humanitarian system has grown rapidly, beyond the empirical description of Glasman’s book. I would like to see ethnographic studies on the deployment of data and actual decision-making in clusters. I am particularly interested in the way REACH^25^ and the International Organization for Migration, as well as data management in OCHA, provide data for the HNRP and other tools. Just as Glasman looked at the Sphere archives and went to Cameroon, we should now do similar studies on data collection and use in these organizations. How do they make their choices in formulating indicators? How do they try to balance sectors and deal with competition among humanitarian organizations? What do they see as the limitations of their work? To what extent can or do they communicate these? How does their data set the incentives for the humanitarian system? Which alternative courses of action are then precluded? But also, in which instances does better use of quantitative and qualitative data help to deal with growing needs and declining resources?

In addition, in new or rapidly evolving crises, humanitarian organizations and donors sometimes apply “no regrets” approaches. They know full well that they do not have enough quantitative and qualitative information yet, but see the urge to spend rapidly in order to prevent further escalation of the suffering. I would like to see more research on the limitations and successes of these approaches. Do they lead to persons in need having a greater say about humanitarian action? Or does decision-making power remain in the hands of humanitarian actors?

Similarly, over the last few years, we have instituted accountability to affected people, the new editions of the *Sphere Handbook* and the Core Humanitarian Standard give accountability a more central role, and OCHA is working on the Flagship Initiative. Is this all really leading to a “redeployment of our infrastructure of commensurability”?^26^ Is this really enabling a humanitarian system that is less donor-oriented, in which persons in need have a higher degree of control of the responses to crises? It is good to have such initiatives, but they are more likely to lead to incremental than wholesale change in the humanitarian system.

**Léa Macias:** Technology has been evolving, with private enterprises entering the field of humanitarian activities and lending their platforms, engineers and algorithms to work on data collected by humanitarian organizations. Both these technological developments and the growth of public–private partnerships between tech companies and humanitarian organization need to be closely looked at. This is at the core of “needology” as it affects the datafication process of people and ultimately their “needs”. The way humanitarian experimentations are being carried out on the justification of an impartial and efficient response is still a field to be continuously explored. From digital identity to algorithms for predicting humanitarian crises, new technologies are reshaping the work of humanitarian organizations, with a real paradigm change from planning a humanitarian response to predicting a humanitarian crisis.

**Bertrand Taithe:** Glasman’s book reflects on historical trends and sets of practices, ways of knowing and forms of understanding. In this sense it is not strictly speaking a commentary on humanitarian aid in 2020 but really a text that engages with some of the most salient technologies of humanitarian practices. I do not think that it claims to be universal in reach or comprehensive. Since 2020 the digital turn has taken on new dimensions, some of which Sandvik studies in her new monograph on humanitarian extractivism.^27^ What she flags up heralds new power imbalances within humanitarian actors and in their use of data. The critique of decision-making processes themselves will have to embrace the way in which artificial intelligence and large datasets will be put to the service of new logics of resource allocation. We are moving to a stage of increasingly opaque algorithms, and that should be a concern for all.

## Figures and Tables

**Table 1:** How explicit normative claims tend to hide implicit causal claims

Humanitarian principles	Humanity	Impartiality		Independence	Neutrality
Explicit normative claim	Secularly sanctioned	Representative claim	Modular technique	Global moral compass	Local moral compass
**Argument**	A right to intervene in this situation (top-down)	No other reason to intervene than to address the needs of the victims (bottom-up)	Aid only addresses the needs of the victims	No impact on the crisis by donor governments and national government	No impact on the crisis
**Implicit causal ** **claim**	Universal permission causality	Compassion causality	Magic bullet causality	Circumscribed causality	Circumscribed causality
**Actual political ** **effect**	**Justification to become active (anywhere)** Conceals the particularity of actual relationships (“partners”) that humanitarian organizations have		**Justification for the actual provision of aid (somewhere)** Conceals the full spectrum of real politics of the provision of aid		

Source: Dennis Dijkzeul, “Inaugural Oration: Humanitarian Studies: Toward a Research Agenda”, Faculty of Social Science, Ruhr University Bochum, 23 June 2010, p. 18 (in mimeo).

## Footnotes

1 Róisín Read, Bertrand Taithe and Roger Mac Ginty, “Data Hubris? Humanitarian Information Systems and the Mirage of Technology”, *Third World Quarterly*, Vol. 37, No. 8, 2016.

2 *Humanitarianism and the Quantification of Human Needs*, p. 9.

3 Dennis Dijkzeul and Claude Iguma Wakenge, “Doing Good, but Looking Bad? Local Perceptions of Two Humanitarian NGOs in the Eastern DRC”, *Disasters*, Vol. 34, No. 4, 2010.

4 *Humanitarianism and the Quantification of Human Needs*, p. 248.

5 *Ibid*., p. 140, citing Margaret L. Satterthwaite, “Indicators in Crisis: Rights-Based Humanitarian Indicators in Post-Earthquake Haiti”, *New York University Journal of International Law and Politics*, Vol. 43, 2011, p. 926.

6 This refers to an apartment on Avenue Giuseppe Motta in Geneva where various early documents of the Sphere Project were stored.

7 *Humanitarianism and the Quantification of Human Needs*, p. 250.

8 Henry Dunant, *A Memory of Solferino*, International Committee of the Red Cross, Geneva, 1986 (first published 1862).

9 Jams Orbinski, *An Imperfect Offering: Humanitarian Action for the Twenty-First Century*, Walker, New York, 2008.

10 See Glasman’s elaboration on this issue in Joël Glasman and Brendan Lawson, “Ten Things We Know about Humanitarian Numbers”, *Journal of Humanitarian Affairs*, Vol. 5, No. 1, 2023.

11 Oliver Bakewell, “Can We Ever Rely on Refugee Statistics?”, *Radical Statistics*, Vol. 72, 1999, available at: www.radstats.org.uk/no072/article1.htm (all internet references were accessed in August 2024).

12 Cormac Ó Gráda, “Making Famine History”, *Journal of Economic Literature*, Vol. 45, No. 1, 2007.

13 Xavier Crombé and Jean-Hervé Jézéquiel (eds), *A Not-So Natural Disaster: Niger 2005*, Hurst, London, 2009.

14 Daniel Maxwell and Nisar Majid, *Famine in Somalia: Competing Imperatives, Collective Failures, 2011*–*12*, Oxford University Press, Oxford, 2016.

15 Susan Sontag, *Regarding the Pain of Others*, Penguin, London, 2003.

16 Fabrice Weissman, *Mortality Emergency Threshold: A Case for Revision*, ALNAP and CRASH, 2018, available at: https://msf-crash.org/en/blog/medicine-and-public-health/mortality-emergency-threshold-case-revision.

17 Cf. Steven Pinker, *Enlightenment Now: The Case for Reason, Science, Humanism, and Progress*, Penguin Random House, London, 2018, pp. 400–409.

18 See also William E. DeMars and Dennis Dijkzeul, “Cómo la teoriá de relaciones internacionales esconde la política de las organizaciones no gubernamentales”, in Laura Zamudio, David Arellano and Jorge Culebro (eds), *Puentes, fronteras y murallas disciplinarias en torno a las organizaciones internacionales*, Centro de Investigación y Docencia Económicas, Mexico City, 2016.

19 As Glasman has stressed elsewhere, colonial indifference is an even greater legacy of colonialism than interference. See his provocative piece “White Saviourism is a Colonial Legacy. But White Indifference is the Larger One”, *The Humanitarian Blog*, 2023, available at: www.ifhv.de/the-humanitarian-blog/WhiteSaviourismisaColonialLegacybutWhiteIndifferenceistheLargerOne.

20 Yan Slobodkin, *The Starving Empire: A History of Famine in France*’*s Colonies*, Cornell University Press, Ithaca, NY, 2023.

21 Alfred W. Crosby, *The Measure of Reality: Quantification and Western Society, 1250*–*1600*, Cambridge University Press, Cambridge, 1997.

22 Nick Couldry and Ulises A. Mejias, *The Costs of Connection: How Data Is Colonizing Human Life and Appropriating It for Capitalism*, Stanford University Press, Stanford, CA, 2019.

23 *Humanitarianism and the Quantification of Human Needs*, p. 249.

24 *Ibid*., p. 249, citing M. L. Satterthwaite, above note 5, pp. 872–873.

25 Along with the International Organization for Migration, REACH is one of the main data providers for the humanitarian system. See the REACH website, available at: www.impact-initiatives.org/what-we-do/reach/.

26 *Humanitarianism and the Quantification of Human Needs*, p. 13.

27 Kristin Bergtora Sandvik, *Humanitarian Extractivism: The Digital Transformation of Aid*, Manchester University Press, Manchester, 2023.

